# Changes in background electroencephalographic activity in benign childhood epilepsy with centrotemporal spikes after oxcarbazepine treatment: a standardized low-resolution brain electromagnetic tomography (sLORETA) study

**DOI:** 10.1186/s12883-018-1228-8

**Published:** 2019-01-03

**Authors:** Ye-Hwa Jun, Tae-Hoon Eom, Young-Hoon Kim, Seung-Yun Chung, In-Goo Lee, Jung-Min Kim

**Affiliations:** 1 Department of Pediatrics, College of Medicine, The Catholic University of Korea, Seoul, Republic of Korea; 2 Department of Internal Medicine, Sanggye Paik Hospital, College of Medicine, Inje University, Seoul, Republic of Korea

**Keywords:** Benign childhood epilepsy with centrotemporal spikes (BCECTS), Benign rolandic epilepsy (BRE), Electroencephalography (EEG), Oxcarbazepine, Standardized low resolution brain electromagnetic tomography (sLORETA)

## Abstract

**Background:**

Several neuroimaging studies have reported neurophysiological alterations in patients with benign childhood epilepsy with centrotemporal spikes (BCECTS). However, reported outcomes have been inconsistent, and the progression of these changes in the brain remains unresolved. Moreover, background electroencephalography (EEG) in cases of BCECTS has not been performed often.

**Methods:**

We investigated background EEG activity changes after six months of oxcarbazepine treatment to better understand the neurophysiological alterations and progression that occur in BCECTS. In 18 children with BCECTS, non-parametric statistical analyses using standardized low resolution brain electromagnetic tomography (sLORETA) were performed to compare the current density distribution of four frequency bands (delta, theta, alpha, and beta) between untreated and treated conditions.

**Results:**

Background EEG activity for the delta frequency band was significantly decreased in the fronto-temporal and limbic regions of the left hemisphere after oxcarbazepine treatment (threshold log-*F*-ratio = ±2.729, *P* < 0.01). The maximum current density difference was found in the parahippocampal gyrus of the left limbic lobe (Montreal Neurological Institute coordinate [x, y, z = 25, − 20, − 10], Brodmann area 28) (log-*F*-ratio = 3.081, *P* < 0.01).

**Conclusions:**

Our results indicate the involvement of the fronto-temporal and limbic cortices in BCECTS, and limbic lobe involvement, including the parahippocampal gyrus, was noted. In addition to evidence of the involvement of the fronto-temporal and limbic cortices in BCECTS, this study also found that an antiepileptic drug could reduce the delta frequency activity of the background EEG in these regions.

## Background

Benign childhood epilepsy with centrotemporal spikes (BCECTS), also known as rolandic epilepsy, is the most common form of benign childhood epilepsy. The seizures are characterized by hemifacial motor seizures, and frequently involve the hand or both the hand and leg on the side ipsilateral to the involved facial side [1]. By definition, centrotemporal spikes observed on electroencephalography (EEG) are the hallmark of BCECTS [2]. The prognosis is usually favorable [1]. Oxcarbazepine was established as an effective initial monotherapy for BCECTS by the Commission of Treatment Guidelines of the International League Against Epilepsy (ILAE) [3].

Several neuroimaging studies using EEG and functional magnetic resonance imaging (MRI) have reported neurophysiological alterations in BCECTS, but the reported outcomes have been inconsistent, and the progress of alterations in the brain remains unresolved. Some studies on dipole source localization have demonstrated that centrotemporal spikes can be reliably modelled by single tangential dipole sources oriented from the central to the frontal lobes and localized in the central regions, namely the rolandic area [4–7]. However, there is growing evidence from neuroimaging studies that BCECTS may functionally and structurally affect a larger portion of the brain [8–12]. Moreover, altered EEG characteristics in BCECTS are mostly studied with respect to qualitative aspects of epileptic activity, whereas background activity in the interictal period is often not quantified [13].

Distributed models for EEG source localization are increasingly sophisticated. Furthermore, a theoretical advantage held by distributed modelling is that its algorithms address the inverse problem with few lead-in assumptions [14]. Standardized low-resolution brain electromagnetic tomography (sLORETA) is one such distributed modelling method. sLORETA can yield 3-dimensional images of electrical neuronal activity, with maximum similarities of orientation and strength between neighbouring neuronal populations [15]. Therefore, it is an excellent tool for investigating the electrophysiological and neuroanatomical features of BCECTS [15–17]. However, few studies have investigated the electrophysiological characteristics of BCECTS using sLORETA [12, 13].

Thus, to better understand the neurophysiological alterations and their development in BCECTS, the present study investigated changes in background EEG activity after six months of oxcarbazepine treatment, which is considered the drug of choice in BCECTS [3]. We performed a comparative analysis of background EEG activity after oxcarbazepine treatment using sLORETA.

## Methods

### Patients and EEG recordings

We retrospectively recruited 18 children with newly diagnosed BCECTS who visited the Division of Pediatric Neurology at Yeouido St. Mary’s Hospital, The Catholic University of Korea, from January 2014 to December 2016 prior to taking medication for epilepsy. The diagnosis of BCECTS was based on the ILAE classification [18].

No diagnostic procedure or treatment was indicated, missed, or postponed for study purposes. The first EEG was recorded with all patients in a drug-free state. The initial oxcarbazepine dose was 10 mg/kg/day, which was progressively increased until the seizures were controlled. A second EEG was performed six months later. At the time of the second EEG, the mean dose of oxcarbazepine was 14.3 mg/kg/day (standard deviation, ±4.7 mg/kg/day). Demographics and clinical features of the participants are summarized in Table 1.

**Table 1:** Demographics and clinical features of patients with benign childhood epilepsy with centrotemporal spikes (BCECTS)

	*N* = 18
Gender (male/female)	11(61.1%) / 7(38.9%)
Handedness (right/left)	17(94.4%) / 1(5.6%)
Age at diagnosis (years)	6.9 ± 1.8 (mean ± SD)
Seizure duration at diagnosis (months)	2.8 ± 3.3 (mean ± SD)
Previous seizure frequency	2.1 ± 1.6 (mean ± SD)
Seizure free with AED	13 (72.2%)
Atypical features	4 (22.2%): ADHD 1, language delay 1, frequent seizures after AED 2, respectively

*SD* standard deviation

*AED* antiepileptic drug

*ADHD* attention deficit hyperactivity disorder

EEG recordings were carried out for 30 min with a Comet® EEG machine (Grass-Telefactor; West Warwick, Rhode Island, United States) at a digitation rate of 200 Hz. Twenty-one silver/silver chloride electrodes were placed according to the International 10–20 EEG system, including the standard 16 temporal and parasagittal scalp sites along with Fz, Cz, Pz, A1, and A2. Additional artifact identification channels were used, including two sites near the eyes, plus respiration and electrocardiography recordings. Eighteen-channel EEG was recorded with a linked ears reference. Additional bipolar derivations were used to differentiate between EEG and eye movement potentials, and to detect electromyographic activity. Impedance did not exceed 5 kΩ. In the EEG derivations, the filters were set at 1.0 and 70 Hz. Sixteen-bit online digitization was used. EEG findings of participants are summarized in Table 2.

**Table 2:** EEG findings of patients with benign childhood epilepsy with centrotemporal spikes (BCECTS)

	*N* = 18
Lateralization of spikes at diagnosis	
left predominant	8 (44.4%)
right predominant	4 (22.2%)
bilateral	6 (33.3%)
Location of maximal negativity at diagnosis	
central	11 (61.1%)
temporal	6 (33.3%)
frontal	1 (6.6%)
Spike index (discharge/min) during AED treatment^*^	
untreated condition	10.6 ± 13.2 (mean ± SD)
treated condition	9.3 ± 15.1 (mean ± SD)

*Paired sample t-test showed no significant difference between the two conditions (P > 0.05)

*EEG* electroencephalography

*AED* antiepileptic drug

*SD* standard deviation

### EEG analysis using sLORETA software

The comparative analysis of background EEG activity was performed using the fast Fourier transform (FFT) technique on EEG recordings, manually segmented 3-s, artifact-free epochs (at rest without non-stationary elements, such as epileptiform or paroxysmal discharges), after visual inspection. This epoch length was adequate to compute an FFT, and short enough to include a sufficient number of artifact-free segments. For each patient, a dataset of 20 epochs was collected for each examination. The segments were representative, as they were (1) chosen randomly across the whole length of the EEG recording and (2) representative of the whole recording. Epochs were selected blindly by one author, and independently reviewed by a second author. These epochs were collected for four frequency bands (delta, 1.5–3.5 Hz; theta, 3.5–7.5 Hz; alpha, 7.5–12.5 Hz; beta, 12.5–25.0 Hz). The limits of each frequency band were referenced from relevant prior studies [19–21]. The EEG recordings were exported into American Standard Code for Information Interchange (ASCII) files, and imported into the sLORETA software.

The current density distributions in the untreated and treated conditions were compared in a voxel-by-voxel analysis of the sLORETA data for four frequency bands. Statistical non-parametric mapping (SnPM) of sLORETA data was used to compare the untreated and treated conditions for four frequency bands [15, 22, 23]. SnPM of sLORETA was performed multiple times with all electrodes or voxels, and for all time samples and discrete frequencies. SnPM of sLORETA images was performed for each contrast with the built-in voxel-wise randomization tests (5000 permutations in the present study) and employing a log-*F*-ratio statistic for dependent groups with a threshold of *P* < 0.01, corrected for multiple comparisons. Correction for multiple comparisons in SnPM with random permutations has been shown to yield results similar to those obtained from statistical parametric mapping with a general linear model with multiple comparison corrections derived from random field theory [22, 23].

In the present study, the critical value was thresholded at *P* < 0.01. With a single threshold test, the statistic image is thresholded at a given critical threshold, and the null hypotheses of voxels with statistic values exceeding this threshold are rejected. Rejection of the null hypothesis occurs if any voxel value exceeds the threshold, a situation clearly determined by calculating the value of the maximum value of the statistic image over the volume of interest. Thus, consideration of the maximum voxel statistic involves the multiple comparisons problem [22]. We adopted a more conservative level of significance (*P* < 0.01), rather than *P* < 0.05, to minimize type 1 errors due to multiple testing [24].

In sLORETA images, the cortex is modelled as a collection of volume elements (6239 voxels, size 5 × 5 × 5 mm), restricted to the cortical grey matter, hippocampus, and amygdala in the digitized Montreal Neurological Institute (MNI) coordinates corrected to the Talairach coordinates [15, 25]. Scalp electrode coordinates on the MNI brain were derived from the International 5% system [26].

## Results

**Fig. 1:**
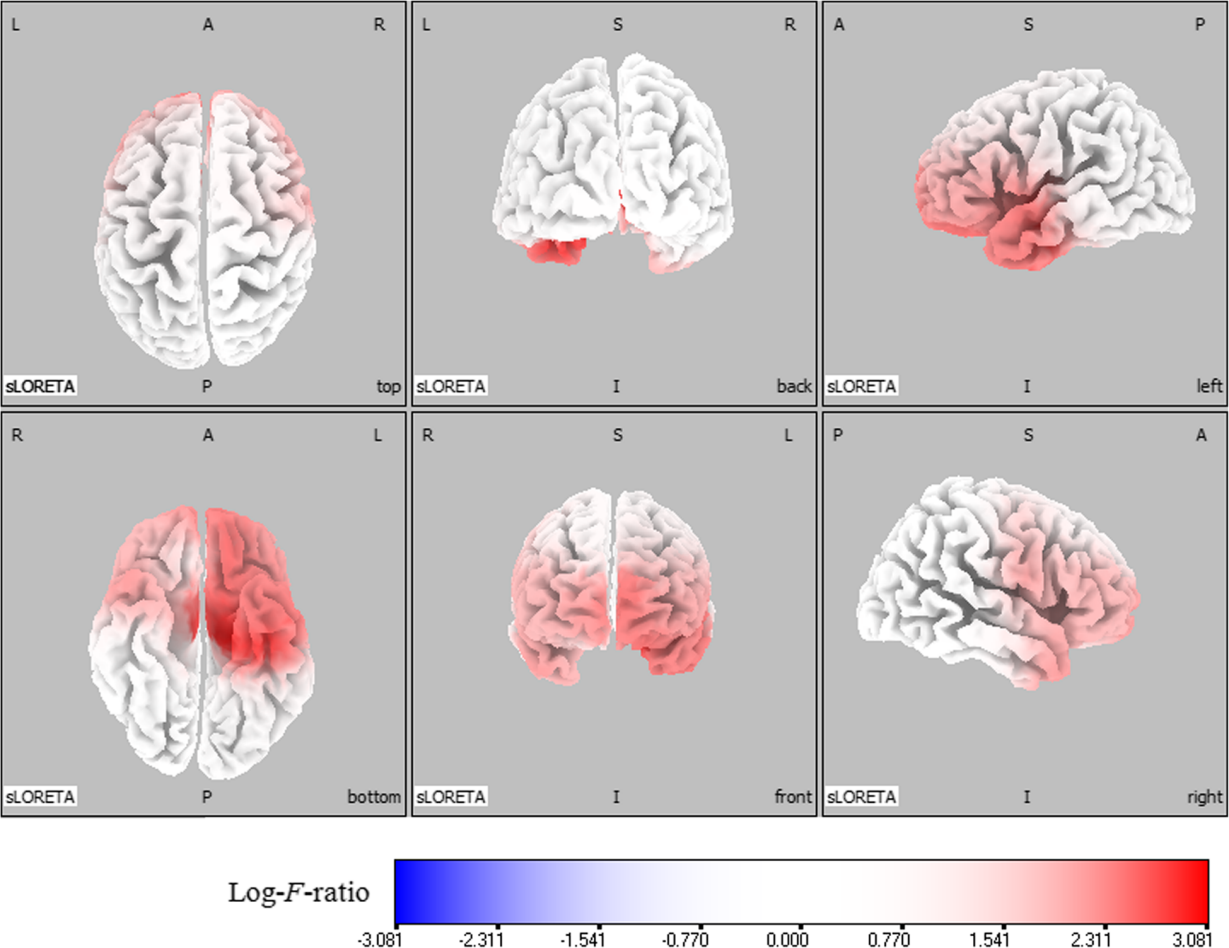
Statistical maps using standardized low resolution brain electromagnetic tomography (sLORETA) of the delta frequency band during oxcarbazepine treatment were projected onto a three-dimensional fiducial brain cortex. Non-parametric statistical analyses were performed to compare the current density distributions between the untreated and treated conditions. Colored areas represent the spatial extent of voxels with a significant difference in the current density. Log-*F*-ratio statistics were employed, and the color scale represents log-*F*-ratio values (threshold log-*F*-ratio = ±2.729, *P* < 0.01). A indicates anterior; *P* posterior, *S* superior, *I* inferior, *L* left, *R* right

**Fig. 2:**
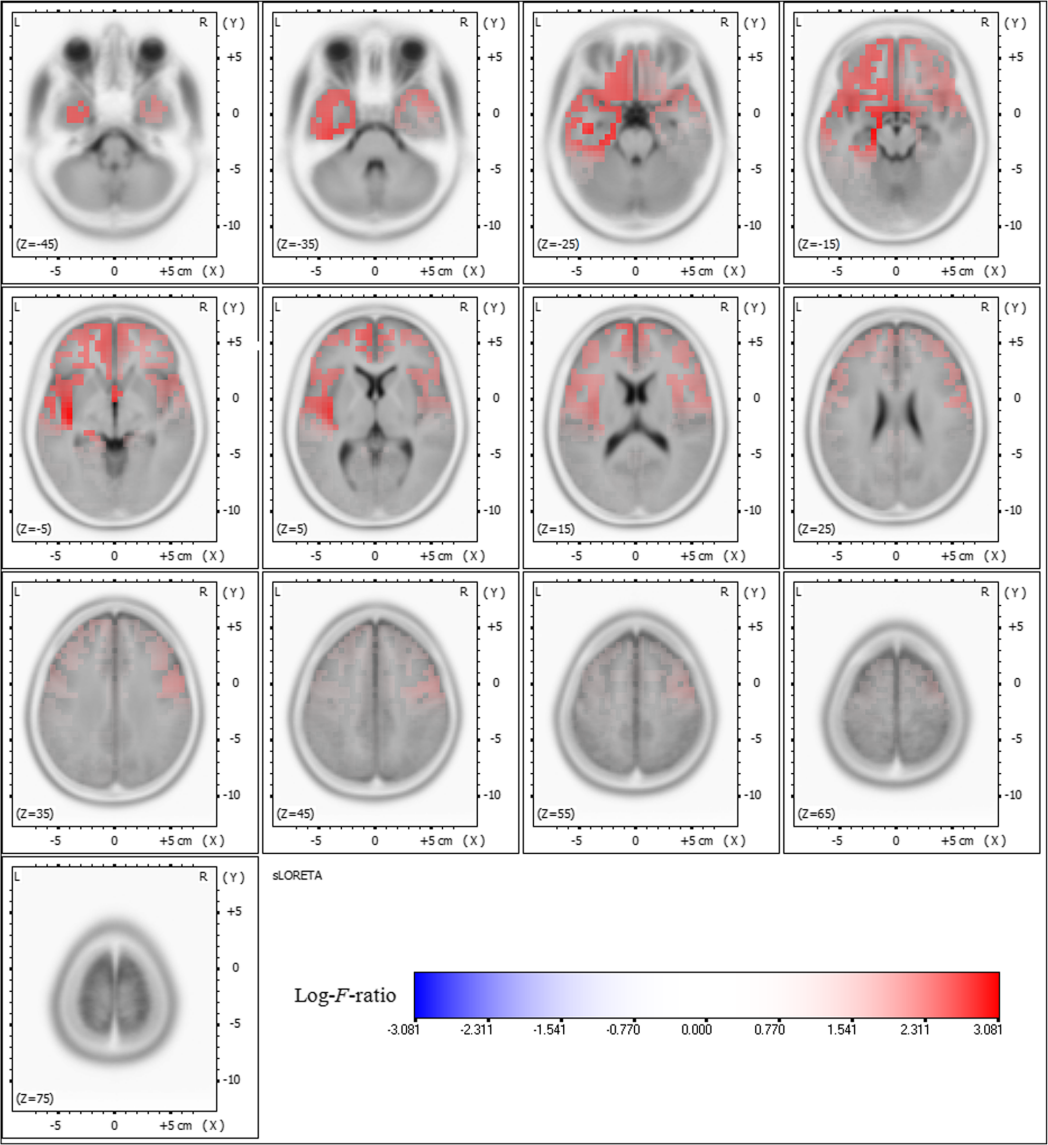
Statistical maps using standardized low resolution brain electromagnetic tomography (sLORETA) of the delta frequency band during oxcarbazepine treatment were projected onto a brain magnetic resonance imaging (MRI) template. Non-parametric statistical analyses were performed to compare the current density distributions between the untreated and treated conditions. Colored areas represent the spatial extent of voxels with a significant difference in the current density. Log-*F*-ratio statistics were employed, and the color scale represents log-*F*-ratio values (threshold log-*F*-ratio = ±2.729, *P* < 0.01). L indicates left; *R* right

Comparative analysis using SnPM of the sLORETA data revealed significant current density differences for the delta frequency band in the frontal lobe (middle frontal gyrus, inferior frontal gyrus, medial frontal gyrus, subcallosal gyrus, rectal gyrus, and orbital gyrus), the temporal lobe (superior temporal gyrus, middle temporal gyrus, inferior temporal gyrus, fusiform gyrus, and subgyral), and the limbic lobe (parahippocampal gyrus and anterior cingulate) of the left hemisphere after six months of oxcarbazepine treatment (threshold log-*F*-ratio = ±2.729, *P* < 0.01) (Figs. 1 and 2). Otherwise, there were no significant differences in the background EEG activity between the untreated and treated conditions.

**Fig. 3:**
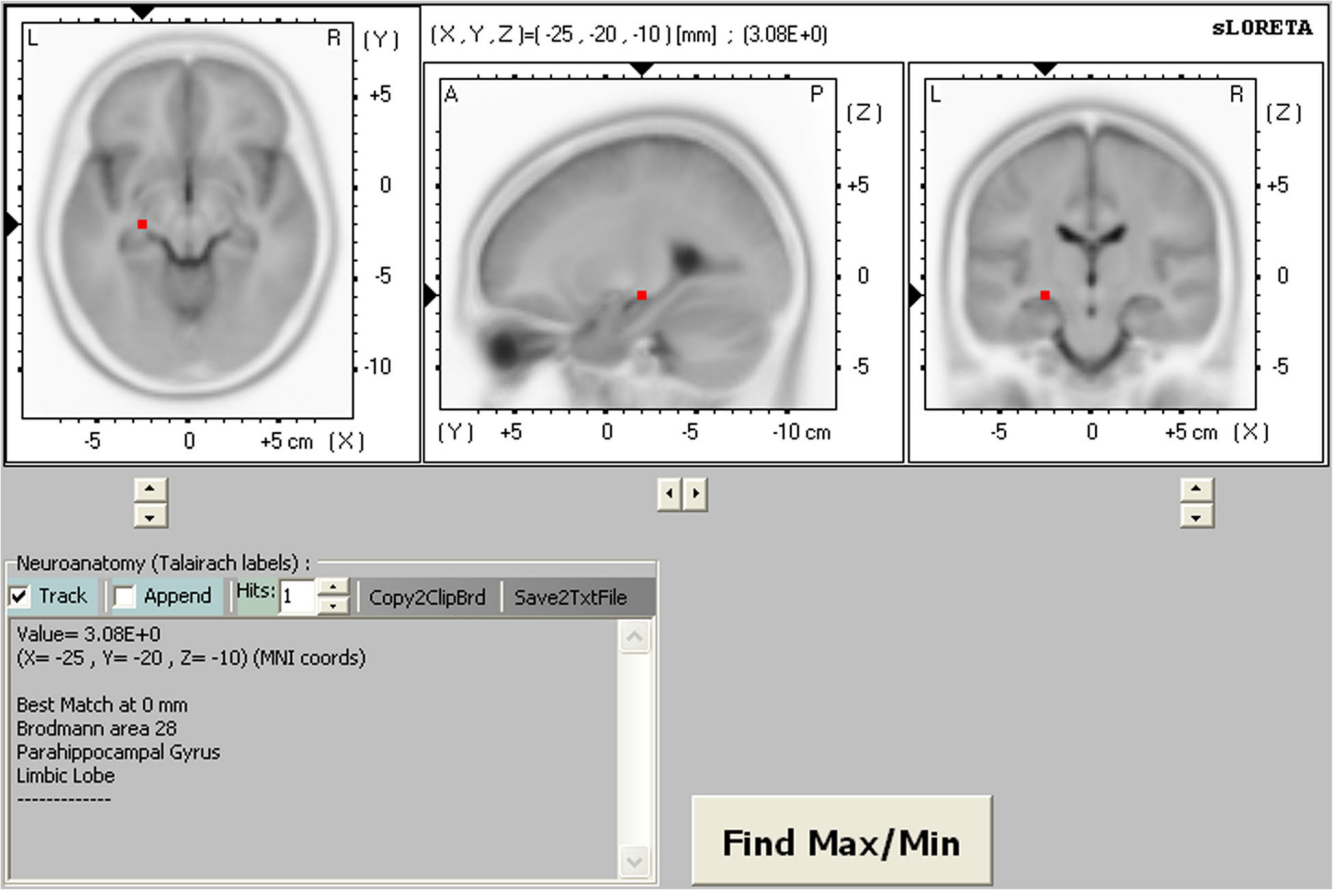
The red-coded area represents voxels with the maximum current density difference after oxcarbazepine treatment located in the parahippocampal gyrus of the left limbic lobe for the delta frequency band (Montreal Neurological Institute coordinate [x, y, z = 25, − 20, − 10], Brodmann area 28) (log-*F*-ratio = 3.081, *P* < 0.01). MNI coord indicates Montreal Neurological Institute coordinate; *A* anterior; *P* posterior, *L* left, *R* right

Figures 1 and 2 show the statistical maps of the spatial extent of voxels within the areas of significant current density differences in the three-dimensional fiducial cortical surface and brain MRI template axial slices, respectively. The significant current density differences for the delta frequency band extend over the frontal, temporal, and limbic cortices of the left hemisphere. As a result, background delta frequency EEG activity after oxcarbazepine treatment was significantly decreased throughout these extended regions. The maximum current density decrease for the delta frequency band was found in the parahippocampal gyrus of the left limbic lobe (MNI coordinate [x, y, z = 25, − 20, − 10], Brodmann area 28) (log-*F*-ratio = 3.081, *P* < 0.01) (Fig. 3).

## Discussion

The essence of our results is that delta frequency activity was significantly reduced in the fronto-temporal and limbic cortices after oxcarbazepine treatment. Several studies have reported enhanced low-frequency band power in the background EEG activity of children with epilepsy compared to that of healthy controls [13, 27, 28]. These results were interpreted as reflecting dysfunction of the relevant cortical regions in children with epilepsy. In addition, later studies showed that antiepileptic drugs reduced background EEG activity in the low-frequency band [20, 29, 30]. These findings are consistent with previous studies. In addition, our results suggest that the fronto-temporal and limbic regions might be involved in BCECTS, and antiepileptic drugs could reduce the low-frequency band power of the background EEG activity in these regions.

The mechanisms underlying these results are not clear, but several possibilities exist. One explanation is that antiepileptic drugs decrease activity in the cortical areas of increased excitability or synchronization that are responsible for seizures [31, 32]. Recent studies have demonstrated that BCECTS was involved in widespread cortical regions over the Rolandic area, but no clearly defined cortical areas have been identified [8–12]. Our previous study using a distributed source model showed that source localization of interictal epileptiform discharges in BCECTS included the central, frontal, and temporal regions surrounding the rolandic area [12]. As mentioned above, several studies have reported enhanced low-frequency activity in the background EEG activity of children with epilepsy [13, 27, 28]. Moreover, increased background EEG activity for the low-frequency band was also demonstrated in the temporal lobe and parietal lobe of BCECTS patients [13]. In an empirical study, oxcarbazepine treatment modified the extent of interictal spikes associated with seizure control [33]. We found the decreased EEG activity seen in those studies closely overlapped regions in our results in the frontal, temporal, parietal, and limbic lobes. This anatomical overlap between the specified cortical areas and the oxcarbazepine effect supports the possibility that oxcarbazepine decreases EEG activity in the cortical neural networks that are involved in BECTS [30].

However, this anatomical distribution cannot be the determinant of the antiepileptic drug effect. Seizure control and epileptogenic activity modification might be linked to decreased EEG activity in the low-frequency band. These observations call for caution in the neurophysiological interpretation of the sLORETA findings, but do not contradict the statement that antiepileptic drugs could decrease background EEG activity in the areas that are involved in BCECTS [30]. The three-dimensional sLORETA results encourage further detailed surveys of our findings. Importantly, further experimental studies may help when interpreting our sLORETA results. For example, a more extended study that includes participants with a range of disease courses undergoing various treatment protocols might answer this question.

It is widely accepted that BCECTS originates from the rolandic area [2, 34]. However, in recent years, widespread cortical involvement in children with BCECTS has been increasingly reported [35–38]. These reports support the view that BCECTS, although initially described as a benign epilepsy syndrome, represents a deviation from normal development during a critical period of brain maturation [36]. BCECTS has been associated with various cognitive function impairments, as well as behavioural problems [1, 2, 13]. Children with BECTS may be more inclined to need antiepileptic drug treatment and special medical attention, instead of the often-applied “wait-and-see” strategy [36].

Our results can be discussed from the lens of epileptogenic zone, seizure-onset zone, symptomatogenic zone, and functional deficit zone. The seizure onset zone is the area of the cortex from which clinical seizures are actually generated, as opposed to the epileptogenic zone, which is the area of the cortex that is indispensable for generation of epileptic seizures. The symptomatogenic zone is the cortical area that is invaded by electrical spread from the seizure-onset zone, and is in the neighbourhood of or directly connected to the seizure-onset zone. The functional deficit zone is the cortical area that does not function normally in the interictal period. The area of functional cortical abnormality frequently extends outside the limits of the epileptogenic zone [39, 40]. However, this area can be defined by a number of tests, including neuropsychological examinations and functional imaging, such as EEG and functional MRI [40]. We did not observe inclusion of the fronto-temporal limbic cortex in the functional deficit zone in this study. Neuropsychological examinations to evaluate the comorbid impairments in children with BCECTS were not routinely included in this study. Further studies including such examinations may provide more data on the functional deficit zone in BCECTS.

An additional limitation of this study is the small number of patients. A larger sample size is desirable to improve the statistical power and generalizability of the results, and would allow the additional possibility of parametric testing [41]. However, the SnPM method adopted here is well suited to the limitations of our study design and sample size [15, 22, 42]. In fact, one of the successful examples of the use of the SnPM method offered by Nichols and Holmes is a functional MRI study between two conditions with 12 subjects, which directly reflects the constraints of the present study [22]. Therefore, we believe that there is a justification for reporting our results. Meanwhile, this study design was not able to make a comparison of the background EEG activity between the untreated patients and age/gender-matched healthy controls. Several previous publications have reported the comparative study of untreated patients with healthy controls for this purpose. Our results are consistent with the previous studies with regards to the affected frequency band, and contribute to further understanding of the progression of brain alterations in BCECTS during antiepileptic drug treatment. A more expanded study with age/gender-matched healthy controls is needed, and such a study might be able to confirm our results. Although sLORETA has no bias location, it tends to show diffused results. sLORETA is an excellent tool with a theoretical advantage of distributed modelling, but some other brain mapping algorithms, including adaptive or Bayesian-based beamformers, for example, Multiple Sparse Priors (MSP) and Champagne, could also be considered. Thus, further studies with larger sample sizes, age−/gender-matched healthy controls, neuropsychological examinations, and other analysis tools are needed to validate and expand on the results of our study.

## Conclusions

In conclusion, this study confirms the involvement of the fronto-temporal and limbic regions in BCECTS, and demonstrates antiepileptic drugs could reduce the delta frequency activity of the background EEG in these regions. It has been suggested that widespread cortical regions over the rolandic area are involved in BCECTS. Our results contribute to further understanding of brain alterations and their progression in BCECTS. In the future, we suggest more expanded studies including a larger population, age−/gender-matched healthy controls, neuropsychological examinations, and other analysis tools to further extend our understanding.

## Abbreviations

BCECTS: Benign childhood epilepsy with centrotemporal spikes
BRE: Benign rolandic epilepsy
EEG: Electroencephalography
FFT: Fast Fourier transform
ILAE: International League Against Epilepsy
MNI: Montreal Neurological Institute
MRI: Magnetic resonance imaging
sLORETA: Standardized low resolution brain electromagnetic tomography
SnPM: Statistical non-parametric mapping
